# SLUA-WSN: Secure and Lightweight Three-Factor-Based User Authentication Protocol for Wireless Sensor Networks

**DOI:** 10.3390/s20154143

**Published:** 2020-07-25

**Authors:** SungJin Yu, YoungHo Park

**Affiliations:** School of Electronics Engineering, Kyungpook National University, Daegu 41566, Korea; darkskiln@knu.ac.kr

**Keywords:** wireless sensor networks, authentication, BAN logic, ROR model, AVISPA simulation

## Abstract

Wireless sensor networks (WSN) are composed of multiple sensor nodes with limited storage, computation, power, and communication capabilities and are widely used in various fields such as banks, hospitals, institutes to national defense, research, and so on. However, useful services are susceptible to security threats because sensitive data in various fields are exchanged via a public channel. Thus, secure authentication protocols are indispensable to provide various services in WSN. In 2019, Mo and Chen presented a lightweight secure user authentication scheme in WSN. We discover that Mo and Chen’s scheme suffers from various security flaws, such as session key exposure and masquerade attacks, and does not provide anonymity, untraceability, and mutual authentication. To resolve the security weaknesses of Mo and Chen’s scheme, we propose a secure and lightweight three-factor-based user authentication protocol for WSN, called SLUA-WSN. The proposed SLUA-WSN can prevent security threats and ensure anonymity, untraceability, and mutual authentication. We analyze the security of SLUA-WSN through the informal and formal analysis, including Burrows–Abadi–Needham (BAN) logic, Real-or-Random (ROR) model, and Automated Verification of Internet Security Protocols and Applications (AVISPA) simulation. Moreover, we compare the performance of SLUA-WSN with some existing schemes. The proposed SLUA-WSN better ensures the security and efficiency than previous proposed scheme and is suitable for practical WSN applications.

## 1. Introduction

Wireless sensor networks (WSN) are widely exploited in terms of enormous applicability [1] and have been used in various fields such as smart homes, smart factories, healthcares, and environmental monitoring [2,3,4,5,6,7,8]. Generally, WSN consist of a gateway node (GWN), a user, and a sensor node (SN) which are resource-limited in smart devices (things, sensors, etc.) [9]. SNs are deployed in various fields and collect a large amount of real-time data. GWN manages data collected by deployed SNs to provide services for legitimate users.

One of the application areas of WSN is a smart home with sensor devices, which provides a better daily life for users [10,11]. A smart home provides various services for users such as automatic checking of the temperature and humidity of the house and controlling light bulbs. However, it may cause serious privacy problems [12,13] because the data collected by SNs are exchanged through a public channel. If data collected by SNs is exposed, a malicious adversary can obtain the private information of users such as daily routines and habits in the house, and also can use the information for criminal purposes. Furthermore, in these application scenarios, smart devices are resource-constrained in terms of computation, communication, and storage overheads, and it is not suitable to apply asymmetric cryptosystems that generate high computational overheads [14]. Therefore, secure and lightweight authentication and key agreement protocols are indispensable to provide secure services for legal users in WSN environments. The secure and lightweight authentication and key agreement protocols must consider the following security requirements.

- Three-factor security: The protocol must meet the three-factor security to protect the legitimate user’s privacy.
- Preventing well-known attacks: The protocol for WSN must be secure against potential attacks, including smart card stolen, masquerade, privileged insider, man-in-the-middle (MITM) attacks, and so on.
- Preventing sensor node capture attack: Even if some sensors are captured by a malicious adversary, it is hard for an adversary to pretend to be other sensors.
- Preventing offline password guessing attack: The protocol must prevent the guessing of the legitimate user’s real password if a malicious adversary either intercepts the transmitted messages or approaches smart card contents.
- Preventing smart card stolen attack: In this attack it is assumed that a malicious adversary can attain the stored secret parameters on the smart card, thus the knowledge of attained parameters should not be enough for the malicious adversary to attain useful information to masquerade a legal user.
- Preventing privileged insider attack: The protocol must be secure to privileged insider attacks where the insider having privileges in the database may access the secret credentials and misuse the contents.
- Anonymity and untraceability: A malicious adversary cannot reveal and trace the real identity of a legitimate user.
- User authentication and key agreement: The protocol must mutually authenticate among entities and successfully establish a secure session key.
- Confidentiality: All transmitted messages communicated between the participants must be safely transmitted using a secret credential so that only legal participants can verify the message.

In 2019, Mo and Chen [15] proposed an elliptic curve cryptosystem (ECC)-based user authentication scheme for WSN. Mo and Chen claimed that their scheme prevents various attacks and provides user anonymity, untraceability, and authentication. However, we prove that their scheme suffers from many drawbacks, including masquerade and replay and session key exposure attacks, and does not provide user anonymity, untraceability, and mutual authentication. In addition, their scheme is not suitable for WSN environments because it requires high communication and computation costs. Consequently, we propose a secure and lightweight three-factor authentication protocol for WSN (SLUA-WSN), considering the efficiency of smart devices and improving the security level of Mo and Chen’s scheme [15].

### 1.1. Contributions and Motivations

The main contributions of our paper can be summarized as follows.

- We propose a secure and lightweight authentication protocol for WSN to resolve the security problems of Mo and Chen’s scheme utilizing secret parameters and biometrics.
- We perform the Burrows–Abadi–Needham (BAN) logic analysis [16] to evaluate that SLUA-WSN ensures secure mutual authentication. We also perform formal security analysis utilizing the Real-or-Random (ROR) model [17] to prove session key security of SLUA-WSN.
- We carry out the simulation analysis using the automated verification of internet security protocols and applications (AVISPA) [18,19] to evaluate that SLUA-WSN prevents against replay and MITM attacks.
- According to the security and performance analysis, we show that the proposed SLUA-WSN achieves better security along with more features, and provides efficient computational, communication, and storage overheads as compared with related schemes.

The motivations of our paper can be summarized as follows.

- Authentication and key agreement protocols for WSN are susceptible to well-known attacks, including sensor node capture, masquerade, and replay attacks.
- Authentication and key agreement protocols for WSN should provide useful convenience for legitimate users and take into account the security requirements.
- Secure and efficient user authentication protocols are essential in WSN, which take into account limitations for resource-constrained smart devices in terms of memory and battery capacity.

We propose a secure and lightweight three-factor authentication protocol for WSN to resolve the security weaknesses of Mo and Chen’s scheme [15]. The proposed SLUA-WSN presents several advantages compared with existing authentication schemes: SLUA-WSN prevents potential attacks, including sensor node capture, replay, privileged insider, and masquerade attacks, and also ensures secure untraceability, user anonymity, and mutual authentication. SLUA-WSN also uses the fuzzy extractor technique to improve the security level of the two-factor-based protocol. Even if two of the three factors are exposed, SLUA-WSN is still secure. Furthermore, SLUA-WSN provides better efficient computation and communication costs with existing schemes because it only uses the hash and XOR operations. Thus, SLUA-WSN is suitable for practical WSN environments because it is more secure and efficient than related schemes.

### 1.2. Organization

The rest of this article is organized as follows. We introduce the related works for WSN environments in Section 2, and present the preliminaries of this paper in Section 3. Section 4 reviews Mo and Chen’s scheme and then Section 5 proves the security shortcomings of Mo and Chen’s scheme. Section 6 presents a secure and lightweight user authentication protocol for WSN environments to enhance the security shortcomings of Mo and Chen’s scheme. Section 7 evaluates the security analysis of SLUA-WSN by performing informal and formal analysis, including BAN logic, ROR model, and AVISPA simulation. Section 8 presents the results of the performance analysis of the SLUA-WSN compared with those of the related schemes. Finally, we conclude the paper in Section 9.

## 2. Related Works

In the last few decades, numerous authentication protocols have been proposed to provide user privacy in the WSN environment [20,21,22,23,24,25]. In 1981, Lamport [26] presented the password-based authentication protocol using a single factor to provide user privacy and anonymity. However, Lamport’s scheme [26] was fragile to offline password guessing attacks because it relied solely on the security of the password. To improve these security problems, Das [27] presented a two-factor authentication scheme using smartcard and password. Das [27] claimed that their scheme is secure and efficient because it uses only hash functions and prevents various attacks. However, some researchers [28,29] pointed out that Das’s scheme [27] has various security drawbacks. Nyang and Lee [28] showed that Das’s scheme [27] is fragile to the sensor node capture and offline password guessing attacks. Nyang and Lee [28] presented a secure authentication scheme in WSN to enhance the security problems of Das’s scheme. In 2010, He et al. [29] proposed a two-factor user authentication scheme for WSN. However, in 2011, Kumar and Lee [30] discovered that He et al.’s scheme [29] cannot provide mutual authentication and generate a session key between each entity. Therefore, these smartcard-based two-factor authentication protocols [27,28,29] were fragile to various attacks.

Numerous biometric-based three-factor authentication protocols have been proposed [31,32,33] to resolve the above-mentioned security issues. Compared with the existing two-factor authentication schemes using a password and smartcard, biometrics (palms, irises, and fingerprints) cannot be stolen or lost because they are very difficult to forget or lose, copy, distribute, guess, break, and forge. Thus, biometric-based three-factor authentication has a higher security level than two-factor authentication.

In recent years, many three-factor authenticated key agreement protocols have been proposed to provide various services in WSN environments [34,35,36]. In 2018, Wu et al. [37] presented a secure three-factor user authentication scheme for WSN. However, in 2019, Mo and Chen [15] demonstrated that if the user inputs an incorrect password at the login process in Wu et al.’s scheme [37], the smartcard does not check whether the password is verified, and the protocol will proceed until GWN finds that the login request of the user was invalid, so GWN performs unnecessary computational resources. In 2017, Wang et al. [38] presented an enhanced three-factor user authentication scheme using ECC for WSN. Unfortunately, Wang et al.’s scheme [38] is susceptible to insider attack because the random nonce for the legitimate user is stored in the database of GWN, and the insider can access and modify it so user login can result in failure. In 2018, Li et al. [39] presented a three-factor-based authentication scheme for WSN in Internet of Things (IoT) environments with adoption of fuzzy extractor to provide high security level. However, Mo and Chen [15] pointed out that Li et al.’s scheme [39] cannot provide three-factor security if the stolen/lost smartcard is obtained by the adversary. In addition, their scheme [39] is not as secure as they claimed because the biometric of the user is collected by the adversary without the awareness of the legitimate user. In 2019, Li et al. [40] presented a secure three-factor-based user authentication protocol for wireless medical sensor networks. However, Mo and Chen [15] demonstrated that their scheme [40] is vulnerable to replay attacks. In 2019, Lu et al. [41] proposed a three-factor authenticated key agreement for WSN using ECC. However, Mo and Chen [15] proved that Lu et al.’s protocol [41] cannot withstand known session-specific temporary information (KSSTI) attacks and cannot provide three-factor security along with session key security. To improve the security drawbacks of Lu et al.’s scheme, Mo and Chen [15] presented a lightweight secure user authenticated key agreement scheme for WSN using ECC. Mo and Chen [15] claimed that their scheme can prevent potential attacks and can ensure anonymity, untraceability, and authentication. However, we analyze that Mo and Chen’s scheme suffers from various security threats, such as session key exposure and masquerade attacks, and cannot ensure anonymity, untraceability, and mutual authentication. In addition, Mo and Chen’s scheme is not practical for WSN because ECC makes the computation and communication overheads burden very heavy. Therefore, we propose a secure and lightweight three-factor user authentication protocol in WSN, considering the efficiency of smart devices and improving security shortcomings of Mo and Chen’s scheme.

## 3. Preliminaries

This section introduces the preliminaries to improve the readability of this paper.

### 3.1. Fuzzy Extractor

This section briefly discusses the concepts of a fuzzy extractor [42]. The fuzzy extractor is a cryptographic method utilizing biometrics to perform secure authentication and it comprises two operations—the generator (Gen) and reproduction (Rep)—which are presented below.

**1.**  Gen: After users imprint the biometric input Bio, Gen generates a consistent random string $\rho \in {\left\{0,1\right\}}^{l}$ and a random auxiliary string $\sigma \in {\left\{0,1\right\}}^{*}$, which is a probabilistic function.
**2.**  Rep: When a noisy biometric $Bio_{new}$ is imprinted, Rep reproduces ρ using value σ, where σ is public reproduction value related with Bio.

### 3.2. Attacker Model

We present the well-known Dolev–Yao (DY) threat model [43] to examine the security of SLUA-WSN. In the DY model, the capabilities of the attacker are as follows.

- Referring to the DY model [43], an attacker can inject, delete, intercept, and eavesdrop the data exchanged over wireless networks.
- A malicious attacker can steal the smart card of legal users and can extract secret credentials stored in memory utilizing power-analysis [44].
- After obtaining the secret credentials of smart card, a malicious attacker may attempt various attacks, including the masquerade, offline password guessing, privileged insider, forward secrecy attacks, and so on [45,46].

### 3.3. System Model

**Figure 1:**
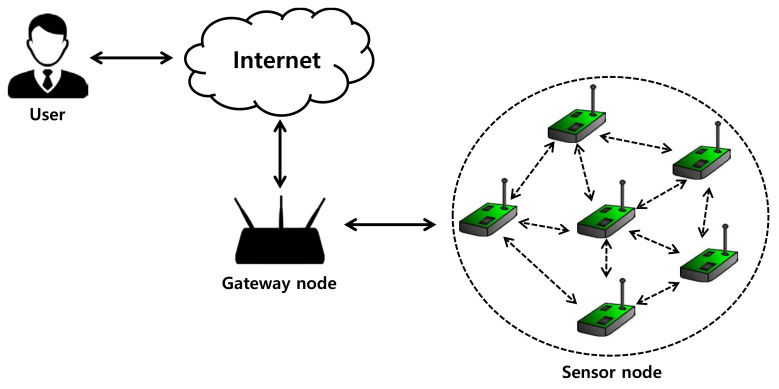
Authentication model in wireless sensor network.

In 2013, Xue et al.’s scheme [47] introduced the five basic authentication mechanism models for WSN. We adopt the first authentication mechanism model presented by Xue et al.’s scheme [47]. This authentication model for WSN consists of three entities: the user, the SN, and the GWN, as shown in Figure 1. Initially, the user contacts GWN to initiate the key agreement between them and the SN. In contrast, the SN checks whether the legitimate user and performs mutual authentication through a GWN. As a result, this model enables mutual authentication between all entities and establishes key agreement between users and corresponding sensor nodes.

## 4. Review of Mo and Chen’s Scheme

**Figure 2:**
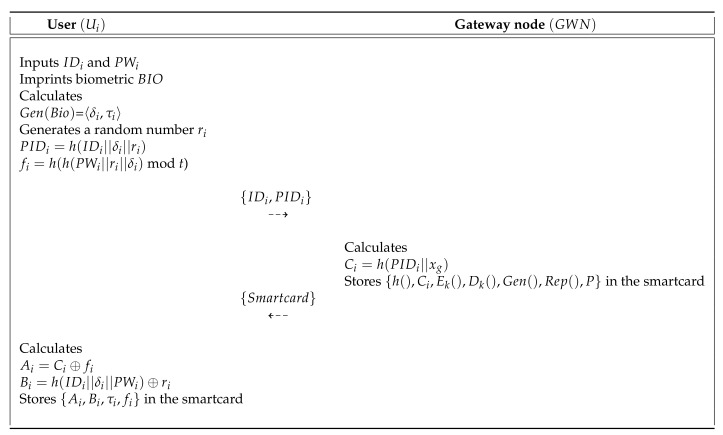
Registration process of Mo and Chen’s scheme.

**Figure 3:**
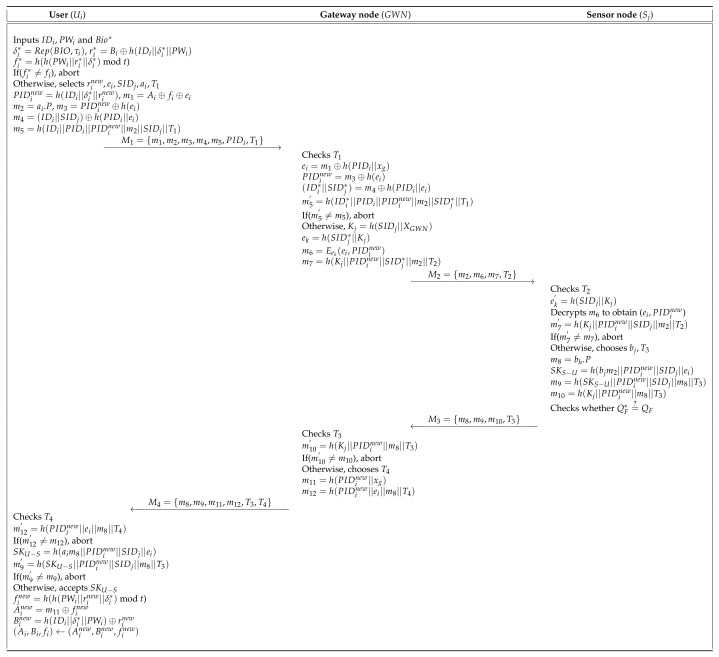
Authentication process of Mo and Chen’s scheme.

Mo and Chen’s scheme [15] presented a secure authentication protocol to provide useful services in WSN. This protocol comprises three entities: the user, the SN, and the GWN. Mo and Chen’s scheme has four processes: pre-deployment, user registration, authentication, and password update. In the pre-deployment process, the gateway node (GWN) selects a unique identity $SID_{j}$ for each sensor ($S_{j}$) and computes $K_{j}=h(SID_{j}\left|\right|X_{GWN})$. Then, GWN sends $\left\{SID_{j},K_{j},P\right\}$ to $S_{j}$ through a secure channel. Finally, $S_{j}$ stores $\left\{SID_{j},K_{j},P\right\}$ in memory. During the user registration process, the GWN issues a smartcard to the legal user who wants to request registration through a secure channel and then helps the agreement of the session key between the $S_{j}$ and the user. They presented a password update process to maintain a high level of security. Figure 2 shows the registration process of Mo and Chen’s scheme, and also the detailed steps involved in the authentication and key agreement process of Mo and Chen’s scheme are as shown in Figure 3. Furthermore, the password update process is described in the following subsections. Table 1 presents the notations used in this paper.

### Password Update Process

If the authorized user requests a new password, Mo and Chen’s scheme can update the password from the gateway as follows.

**Step** **1:** $U_{i}$ inputs $ID_{i}$ and the old $PW_{i}$ and imprints $Bio^{*}$, and inserts the smartcard (SC) in the reader. After that, the SC calculates $Gen\left(Bio^{*}\right)=\left(\delta_{i}^{*},\tau_{i}^{*}\right)$, $r_{i}^{*}=B_{i}⊕h(ID_{i}\left|\right|\delta_{i}^{*}||PW_{i})$, and $f_{i}^{{}^{\prime }}=h(h(ID_{i}\left|\right|r_{i}^{*}||PW_{i})$ mod *t* and checks whether $f_{i}^{{}^{\prime }}\overset{?}{=}f_{i}$ holds. If the condition is false, the communication is aborted.
**Step** **2:** $U_{i}$ inputs a new $PW_{i}^{new}$, computes $f_{i}^{new}=h(h(PW_{i}^{new}\left|\right|r_{i}^{*}\left|\right|\delta_{i}^{*})$ mod *t*, $A_{i}^{new}=C_{i}⊕f_{i}^{new}$, $B_{i}^{new}=h(ID_{i}\left|\right|\delta_{i}^{*}||PW_{i}^{new})⊕r_{i}^{*}$ and replaces ($A_{i},B_{i},f_{i}$) with ($A_{i}^{new},B_{i}^{new},f_{i}^{new}$).

## 5. Security Flaws of Mo and Chen’s Scheme

We discuss the security flaws of Mo and Chen’s scheme, including session key exposure and masquerade attacks. Furthermore, we discover that Mo and Chen’s scheme cannot ensure user anonymity, untraceability, and mutual authentication.

### 5.1. Masquerade Attack

In this attack, a malicious attacker (MA) may attempt to impersonate legal users through stolen smartcard. According to Section 3.2, we assume that MA is able to extract the secret credentials $\left\{A_{i},B_{i},\tau_{i},f_{i}\right\}$ stored in the smart card. Furthermore, MA can intercept the messages exchanged over the wireless network. Therefore, MA can perform the masquerade attack as shown in the following detailed steps.

**Step** **1:** A MA first calculates $e_{i}=m_{1}⊕A_{i}⊕f_{i}$, $PID_{i}^{new}=m_{3}⊕h\left(e_{i}\right)$, $(ID_{i}||SID_{j})=m_{4}⊕h(PID_{i}\left|\right|e_{i})$, and $m_{5}=h(ID_{i}||PID_{i}||PID_{i}^{new}\left|\right|m_{2}||SID_{j}\left|\right|T_{1})$. After that, the MA generates the two random numbers $e_{MA}$, $a_{MA}$ and computes $m_{1MA}=A_{i}⊕f_{i}⊕e_{MA}$, $m_{2MA}=a_{MA}P$, $m_{3MA}=PID_{i}^{new}⊕h\left(e_{MA}\right)$, $m_{4MA}=(ID_{i}||SID_{j})⊕h(PID_{i}\left|\right|e_{MA})$ and $m_{5MA}=h(ID_{i}||PID_{i}||PID_{i}^{new}\left|\right|m_{2}||SID_{j}\left|\right|T_{1})$. The MA sends $M_{1}=\left\{m_{1MA},m_{2MA},m_{3MA},m_{4MA},m_{5MA},PID_{i},T_{1}\right\}$ to the GWN over wireless networks.
**Step** **2:** Upon getting the $M_{1}$, the GWN verifies the validity of $T_{1}$. If it is equal, the GWN computes $e_{MA}=m_{1MA}⊕h(PID_{i}\left|\right|x_{g})$, $PID_{i}^{new}=m_{3MA}⊕h\left(e_{MA}\right)$, $(ID_{i}^{{}^{\prime }}||SID_{j}^{{}^{\prime }})=m_{4MA}⊕h(PID_{i}\left|\right|e_{MA})$, and $m_{5MA}=h(ID_{i}^{{}^{\prime }}||PID_{i}||PID_{i}^{new}\left|\right|m_{2MA}||SID_{j}^{{}^{\prime }}\left|\right|T_{1})$. Then, the GWN checks $m_{5MA}^{{}^{\prime }}\overset{?}{=}m_{5MA}$. If it is correct, the GWN computes $e_{k}=h(SID_{j}^{{}^{\prime }}\left|\right|K_{j})$, $m_{6}=E_{e_{k}}\left(e_{MA},PID_{i}^{new}\right)$ and $m_{7}=h(K_{j}||PID_{i}^{new}||SID_{j}^{{}^{\prime }}\left|\right|m_{2}\left|\right|T_{2})$. Next, the GWN sends $M_{2}=\left\{m_{2MA},m_{6},m_{7},T_{2}\right\}$ to the $S_{j}$.
**Step** **3:** After getting the $M_{2}$, the $S_{j}$ verifies the $T_{2}$. If it is equal, the $S_{j}$ calculates $e_{k}^{{}^{\prime }}=h(SID_{j}\left|\right|K_{j})$ and decrypts $m_{6}$ to get ($e_{MA},PID_{i}^{new}$). After that, the $S_{j}$ calculates $m_{7}^{{}^{\prime }}=h(K_{j}||PID_{i}^{new}||SID_{j}\left|\right|m_{2}\left|\right|T_{2})$ and then checks $m_{7}^{{}^{\prime }}\overset{?}{=}m_{7}$. If the condition is equal, the $S_{j}$ selects a random number $b_{j}$ and timestamp $T_{3}$. Then, $S_{j}$ computes $m_{8}=b_{j}P$, $SK_{S-MA}=h(b_{j}m_{2MA}||PID_{i}^{new}||SID_{j}\left|\right|e_{MA})$, $m_{9MA}=h(SK_{S-MA}||PID_{i}^{new}||SID_{j}\left|\right|m_{8}\left|\right|T_{3})$ and $m_{10}=h(K_{j}||PID_{i}^{new}\left|\right|m_{8}\left|\right|T_{3})$. Finally, $S_{j}$ sends $M_{3}=\left\{m_{8},m_{9MA},m_{10},T_{3}\right\}$ to the GWN.
**Step** **4:** Upon getting the $M_{3}$, the GWN verifies the validity of $T_{3}$. If the condition is equal, the GWN calculates $m_{10}^{{}^{\prime }}=h(K_{j}||PID_{i}^{new}\left|\right|m_{8}\left|\right|T_{3})$ and verifies $m_{10}^{{}^{\prime }}\overset{?}{=}m_{10}$. If the condition is valid, the GWN selects $T_{4}$ and calculates $m_{11}=h(PID_{i}^{new}\left|\right|x_{g})$ and $m_{12MA}=h(PID_{i}^{new}\left|\right|e_{MA}\left|\right|m_{8}\left|\right|T_{4})$. Finally, GWN sends $M_{4}=\left\{m_{8},m_{9MA},m_{11},m_{12MA},T_{3},T_{4}\right\}$ to the $U_{i}$.
**Step** **5:** After getting the $M_{4}$, the MA checks the $T_{4}$ and calculates $m_{12MA}^{{}^{\prime }}=h(PID_{i}^{new}\left|\right|e_{MA}\left|\right|m_{8}\left|\right|T_{4})$ and checks $m_{12MA}^{{}^{\prime }}\overset{?}{=}m_{12MA}$. If it is equal, the MA computes $SK_{MA-S}=h(a_{MA}\left|\right|m_{8}||PID_{i}^{new}||SID_{j}\left|\right|e_{MA})$ and $m_{9}^{{}^{\prime }}=h(SK_{MA-S}||PID_{i}^{new}||SID_{j}\left|\right|m_{8}\left|\right|T_{3})$.

As a result, Mo and Chen’s scheme cannot prevent the masquerade attack because the MA can impersonate an legitimate user successfully.

### 5.2. Session Key Exposure Attack

In Mo and Chen’s scheme, they claimed that their scheme could prevent to session key exposure attack because a MA could not obtain the secret credentials. However, according to Section 5.1, we prove that MA is able to impersonate legal users $U_{i}$ and calculates the session key SK as follows. Referring to Section 3.2, the MA can extract secret credentials $\left\{A_{i},B_{i},\tau_{i},f_{i}\right\}$ stored in the smartcard. Then, the MA is able to intercept the exchanged messages between $U_{i}$, GWN, and $S_{j}$ via wireless networks. If so, the MA can calculate $e_{i}$, $PID_{i}^{new}$ and $\left(ID_{i}||SID_{j}\right)$. After that, the MA selects random numbers $e_{MA},a_{MA}$ and can successfully generate new messages $\left\{m_{1MA},m_{2MA},m_{3MA},m_{4MA},m_{5MA}\right\}$ by utilizing $e_{MA}$ and $a_{MA}$. Consequently, the MA can successfully perform the session key exposure attack by calculating $SK_{MA-S}=h(a_{MA}\left|\right|m_{8}||PID_{i}^{new}||SID_{j}\left|\right|e_{MA})$ and disguise as legitimate users.

### 5.3. Anonymity and Untraceability

Referring to Section 5.1, the MA can trace a legitimate user $U_{i}$ and can obtain the real identities $\left\{ID_{i},SID_{j}\right\}$ of $U_{i}$ and $S_{j}$. The MA computes $e_{i}=m_{1}⊕A_{i}⊕f_{i}$ utilizing secret credentials $\left\{A_{i},f_{i}\right\}$ stored in the smart card. After that, the MA can compute $(ID_{i}||SID_{j})=m_{4}⊕h(PID_{i}\left|\right|e_{i})$, $PID_{i}^{new}=m_{3}⊕h\left(e_{i}\right)$, and $m_{5}=h(ID_{i}||PID_{i}||PID_{i}^{new}\left|\right|m_{2}||SID_{j}\left|\right|T_{1})$ successfully. Thus, Mo and Chen’s scheme does not ensure user anonymity and untraceability.

### 5.4. Mutual Authentication

Mo and Chen’s scheme asserted that their scheme provides secure mutual authentication among the $U_{i}$, GWN, and $S_{j}$. However, referring to Section 5.1, the MA can generate authentication request message $m_{5MA}=h(ID_{i}||PID_{i}||PID_{i}^{new}\left|\right|m_{2}||SID_{j}\left|\right|T_{1})$, response message $m_{12MA}=h(PID_{i}^{new}\left|\right|e_{MA}\left|\right|m_{8}\left|\right|T_{4})$, and then can calculate session key $SK_{MA-S}=h(a_{MA}\left|\right|m_{8}||PID_{i}^{new}||SID_{j}\left|\right|e_{MA})$. As a result, we prove that their scheme cannot provide correct mutual authentication among $U_{i}$, GWN, and $S_{j}$.

## 6. Proposed Scheme

We present a secure and lightweight user authentication protocol in WSN to improve the security flaws of [15]. The proposed SLUA-WSN comprises the same process as that Mo and Chen’s scheme. The details of the four processes are shown below.

### 6.1. Pre-Deployment Process

**Figure 4:**
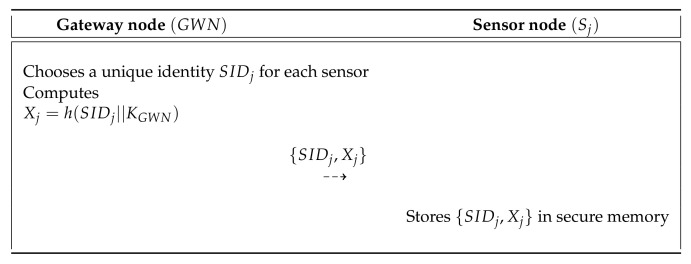
Pre-deployment process of the proposed scheme.

This process is similar to the pre-deployment process given in Mo and Chen’s scheme [15]. In Figure 4, we show the user registration process of SLUA-WSN and the detailed steps are below.

**Step** **1:** GWN selects a unique identity $SID_{j}$ for sensors and computes $X_{j}=h(SID_{j}\left|\right|K_{GWN})$. Finally, GWN sends $\left\{SID_{j},X_{j}\right\}$ to the $S_{j}$ over a secure communication.
**Step** **2:** Upon receiving the messages, the $S_{j}$ stores them in secure memory.

### 6.2. User Registration Process

**Figure 5:**
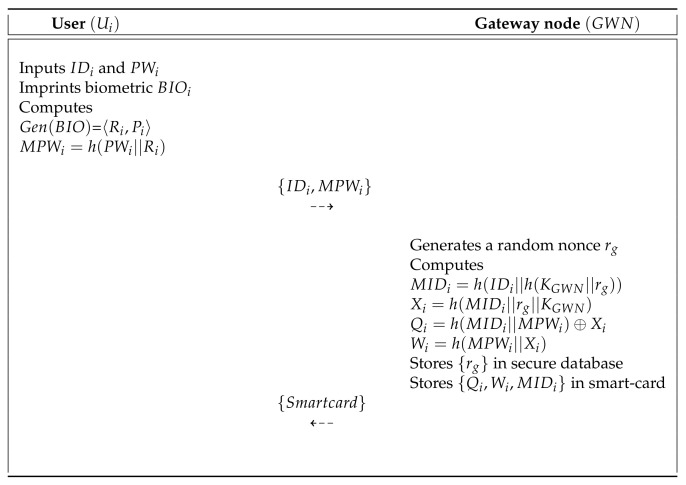
User registration process of our scheme.

The $U_{i}$ must register within GWN to access various services. In Figure 5, we show the user registration process of SLUA-WSN and the detailed steps are below.

**Step** **1:** $U_{i}$ inputs the $ID_{i}$ and $PW_{i}$ and imprints biometric $BIO_{i}$. Then, the $U_{i}$ computes Gen(BIO)=$\langle R_{i},P_{i}\rangle$ and $MPW_{i}=h(PW_{i}\left|\right|R_{i})$, and sends $\left\{ID_{i},MPW_{i}\right\}$ to the GWN over a secure communication.
**Step** **2:** After reception of messages, the GWN generates a random nonce $r_{g}$ and calculates $MID_{i}=h(ID_{i}||h(K_{GWN}\left|\right|r_{g}))$, $X_{i}=h(MID_{i}\left|\right|r_{g}\left|\right|K_{GWN})$, $Q_{i}=h(MID_{i}||MPW_{i})⊕X_{i}$ and $W_{i}=h(MPW_{i}\left|\right|X_{i})$, and then stores $\left\{r_{g}\right\}$ in secure database. After that, the GWN stores $\left\{Q_{i},W_{i},MID_{i}\right\}$ in the smart card and issues it to the $U_{i}$.

### 6.3. Authentication Process

**Figure 6:**
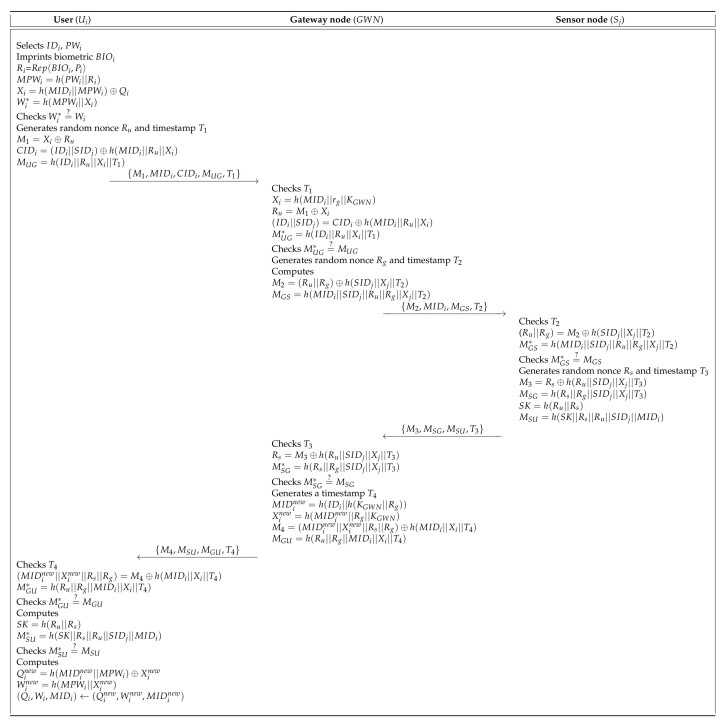
Authentication process of our scheme.

After performing the registration process, the registered $U_{i}$ requests authentication to the GWN in order to establish the session key. In Figure 6, we show the authentication process of SLUA-WSN and the detailed steps are below.

**Step** **1:** $U_{i}$ first inserts the smart card and inputs $ID_{i}$ and $PW_{i}$. Then, the $U_{i}$ imprints $BIO_{i}$ and computes $R_{i}$=$Rep\langle BIO_{i},P_{i}\rangle$, $MPW_{i}=h(PW_{i}\left|\right|R_{i})$, $X_{i}=h(MID_{i}||MPW_{i})⊕Q_{i}$, and $W_{i}^{*}=h(MPW_{i}\left|\right|X_{i})$, and then checks $W_{i}^{*}\overset{?}{=}W_{i}$. If the condition is valid, the $U_{i}$ generates a random nonce $R_{u}$ and a timestamp $T_{1}$. The $U_{i}$ computes $M_{1}=X_{i}⊕R_{u}$, $CID_{i}=(ID_{i}||SID_{j})⊕h(MID_{i}\left|\right|R_{u}\left|\right|X_{i})$, and $M_{UG}=h(ID_{i}\left|\right|R_{u}\left|\right|X_{i}\left|\right|T_{1})$, and sends $\left\{M_{1},MID_{i},CID_{i},M_{UG},T_{1}\right\}$ to the GWN over an insecure channel.
**Step** **2:** Upon reception of messages, the GWN checks the validity of $T_{1}$ and calculates $X_{i}=h(MID_{i}\left|\right|r_{g}\left|\right|K_{GWN})$, $R_{u}=M_{1}⊕X_{i}$, $(ID_{i}||SID_{j})=CID_{i}⊕h(MID_{i}\left|\right|R_{u}\left|\right|X_{i})$ and $M_{UG}^{*}=h(ID_{i}\left|\right|R_{u}\left|\right|X_{i}\left|\right|T_{1})$ and then, checks $M_{UG}^{*}\overset{?}{=}M_{UG}$. If the condition is correct, the GWN calculates $M_{2}=(R_{u}\left|\right|R_{g})⊕h(SID_{j}\left|\right|X_{j}\left|\right|T_{2})$ and $M_{GS}=h(MID_{i}||SID_{j}\left|\right|R_{u}\left|\right|R_{g}\left|\right|X_{j}\left|\right|T_{2})$, and sends $\left\{M_{2},MID_{i},M_{GS},T_{2}\right\}$ to the $S_{j}$.
**Step** **3:** After reception of messages, the $S_{j}$ checks the validity of $T_{2}$ and computes $(R_{u}\left|\right|R_{g})=M_{2}⊕h(SID_{j}\left|\right|X_{j}\left|\right|T_{2})$ and $M_{GS}^{*}=h(MID_{i}||SID_{j}\left|\right|R_{u}\left|\right|R_{g}\left|\right|X_{j}\left|\right|T_{2})$ and checks $M_{GS}^{*}\overset{?}{=}M_{GS}$. If it is valid, the $S_{j}$ generates a random nonce $R_{s}$ and timestamp $T_{3}$ and calculates $M_{3}=R_{s}⊕h(R_{u}||SID_{j}\left|\right|X_{j}\left|\right|T_{3})$, $M_{SG}=h(R_{s}\left|\right|R_{g}||SID_{j}\left|\right|X_{j}\left|\right|T_{3})$, $SK=h(R_{u}||R_{s})$, and $M_{SU}=h(SK||R_{s}\left|\right|R_{u}||SID_{j}||MID_{i})$, and then sends $\left\{M_{3},M_{SG},M_{SU},T_{3}\right\}$ to the GWN over an insecure channel.
**Step** **4:** Upon reception of messages, the GWN checks the validity of $T_{3}$ and calculates $R_{s}=M_{3}⊕h(R_{u}||SID_{j}\left|\right|X_{j}\left|\right|T_{3})$ and $M_{SG}^{*}=h(R_{s}\left|\right|R_{g}||SID_{j}\left|\right|X_{j}\left|\right|T_{3})$, and checks $M_{SG}^{*}\overset{?}{=}M_{SG}$. If it is valid, the GWN generates a timestamp $T_{4}$ and computes $MID_{i}^{new}=h(ID_{i}||h(K_{GWN}\left|\right|R_{g}))$, $X_{i}^{new}=h(MID_{i}^{new}\left|\right|R_{g}\left|\right|K_{GWN})$, $M_{4}=(MID_{i}^{new}\left|\right|X_{i}^{new}\left|\right|R_{s}\left|\right|R_{g})⊕h(MID_{i}\left|\right|X_{i}\left|\right|T_{4})$, and $M_{GU}=h(R_{u}\left|\right|R_{g}||MID_{i}\left|\right|X_{i}\left|\right|T_{4})$ and sends $\left\{M_{4},M_{SU},M_{GU},T_{4}\right\}$ to the $U_{i}$.
**Step** **5:** After reception of messages, the $U_{i}$ checks the validity of $T_{4}$ and computes $(MID_{i}^{new}\left|\right|X_{i}^{new}\left|\right|R_{s}\left|\right|R_{g})=M_{4}⊕h(MID_{i}\left|\right|X_{i}\left|\right|T_{4})$ and $M_{GU}^{*}=h(R_{u}\left|\right|R_{g}||MID_{i}\left|\right|X_{i}\left|\right|T_{4})$, and then checks $M_{GU}^{*}\overset{?}{=}M_{GU}$. If the condition is valid, the $U_{i}$ computes $SK=h(R_{u}||R_{s})$ and $M_{SU}^{*}=h(SK||R_{s}\left|\right|R_{u}||SID_{j}||MID_{i})$, and checks $M_{SU}^{*}\overset{?}{=}M_{SU}$. If the condition is correct, the $U_{i}$ computes $Q_{i}^{new}=h(MID_{i}^{MPW}||MPW_{i})⊕X_{i}^{new}$, and $W_{i}^{new}=h(MPW_{i}\left|\right|X_{i}^{new})$ and replaces $\left\{Q_{i},W_{i},MID_{i}\right\}$ with $\left\{Q_{i}^{new},W_{i}^{new},MID_{i}^{new}\right\}$. Consequently, the $U_{i}$, the GWN and $S_{j}$ are mutually authenticated successfully.

### 6.4. Password Change Process

In SLUA-WSN, an authorized $U_{i}$ can freely update their password. The detailed steps of the password change process are below.

**Step** **1:** $U_{i}$ inputs $ID_{i}^{{}^{\prime }}$ and $PW_{i}^{{}^{\prime }}$ and imprints biometric $BIO_{i}^{{}^{\prime }}$. After that, the $U_{i}$ computes $Gen(BIO^{{}^{\prime }})$=$\langle R_{i}^{{}^{\prime }},P_{i}^{{}^{\prime }}\rangle$ and $MPW_{i}^{{}^{\prime }}=h(PW_{i}^{{}^{\prime }}\left|\right|R_{i}^{{}^{\prime }})$ and then sends $\left\{ID_{i}^{{}^{\prime }},MPW_{i}^{{}^{\prime }}\right\}$ to the SC over a secure communication.
**Step** **2:** Upon reception of messages, the SC calculates $X_{i}^{{}^{\prime }}=Q_{i}^{{}^{\prime }}⊕h(MID_{i}^{{}^{\prime }}||MPW_{i}^{{}^{\prime }})$ and $W_{i}^{{}^{\prime }}=h(MPW_{i}^{{}^{\prime }}\left|\right|X_{i}^{{}^{\prime }})$ and sends authentication message to the $U_{i}$.
**Step** **3:** After reception of messages, the $U_{i}$ chooses a new $PW_{i}^{new}$ and imprints a new $BIO^{new}$. Then, the $U_{i}$ calculates $Gen(BIO^{new})$=$\langle R_{i}^{new},P_{i}^{new}\rangle$ and $MPW_{i}^{new}=h(PW_{i}^{new}\left|\right|R_{i}^{new})$ and sends $\left\{MPW_{i}^{new}\right\}$ to the SC over a secure channel.
**Step** **4:** Upon reception of messages, the SC calculates $Q_{i}^{new}=h(MID_{i}^{{}^{\prime }}||MPW_{i}^{new})⊕X_{i}^{{}^{\prime }}$ and $W_{i}^{new}=h(MPW_{i}^{new}\left|\right|X_{i}^{{}^{\prime }})$ and then replaces $\left\{Q_{i}^{{}^{\prime }},W_{i}^{{}^{\prime }}\right\}$ with $\left\{Q_{i}^{new},W_{i}^{new}\right\}$ successfully.

## 7. Security Analysis

This section assessed the security of SLUA-WSN by using informal and formal security analysis such as BAN logic, ROR model, and AVISPA simulation, which are widely known security models.

### 7.1. Informal Security Analysis

The security of SLUA-WSN is assessed by performing an informal security analysis. We show that SLUA-WSN can resist potential security threats, including masquerade, sensor node capture, replay, and privileged insider attacks, and ensure secure authentication and anonymity.

#### 7.1.1. Masquerade Attack

In this attack, the MA attempts to masquerade a legitimate user by intercepting messages transmitted over an insecure channel. However, the MA cannot generate the request messages $\left\{M_{1},MID_{i},CID_{i},M_{UG}\right\}$ in the proposed SLUA-WSN correctly. The MA cannot compute the request messages because MA cannot get $U_{i}$’s real identity $ID_{i}$, the biometric BIO, and the random nonce $R_{u}$. As a result, SLUA-WSN resists masquerade attacks.

#### 7.1.2. Replay Attack

Assuming that the MA attempts the replay attack utilizing previously exchanged data over an insecure channel, even if the MA intercepts the request message $\left\{M_{1},MID_{i},CID_{i},M_{UG},T_{1}\right\}$ in the previous session, the proposed SLUA-WSN verifies the freshness of the timestamp. In addition, the request messages are protected with secret parameter $X_{i}$ and random nonce $R_{u}$. Thus, SLUA-WSN prevents replay attacks.

#### 7.1.3. Sensor Node Capture Attack

As sensor nodes are typically placed in unmanned or hostile areas, the MA can easily capture sensor nodes. However, each $S_{j}$ has a unique $SID_{j}$ and a secret parameter $X_{j}$. Even if some sensor nodes are captured by the MA, it is difficult to impersonate that the MA is another sensor. Therefore, the MA does not have any ability to compromise other SK established between the $U_{i}$ and non-compromised $S_{j}$. Thus, SLUA-WSN prevents sensor node capture attacks.

#### 7.1.4. Privileged Insider Attack

In this attack, the privileged insider is able to access the password of the user stored in GWN and disguises the user to log in to other systems. However, the user in the proposed SLUA-WSN only sends $\left\{ID_{i},MPW_{i}\right\}$ to the GWN during the registration process. Consequently, SLUA-WSN prevents privileged insider attacks because the privileged insider cannot obtain the real password of the legitimate user.

#### 7.1.5. Anonymity and Untraceability

We assume that the MA can extract secret credentials stored in a smartcard and is able to eavesdrop the message exchanged in each session. However, the MA cannot trace a legal user $U_{i}$ because all exchanged messages are updated every session, and also $\left\{Q_{i},W_{i},MID_{i}\right\}$ messages in the proposed SLUA-WSN update with $\left\{Q_{i}^{new},W_{i}^{new},MID_{i}^{new}\right\}$. Moreover, the MA cannot obtain the real $ID_{i}$ of $U_{i}$ because it is masked with XOR and hash functions. Thus, SLUA-WSN provides anonymity and untraceability because the MA cannot retrieve $ID_{i}$ without knowing a secret parameter $X_{i}$ and a random nonce $R_{u}$.

#### 7.1.6. Mutual Authentication

In SLUA-WSN, each entity performs mutual authentication successfully. Upon getting the authentication request messages $\left\{M_{1},MID_{i},CID_{i},M_{UG}\right\}$ from the $U_{i}$, the GWN verifies $M_{UG}^{*}\overset{?}{=}M_{UG}$. If the condition is correct, the GWN authenticates the $U_{i}$. After getting the messages $\left\{M_{2},MID_{i},M_{GS},T_{2}\right\}$ from the GWN, the $S_{j}$ checks $M_{GS}^{*}\overset{?}{=}M_{GS}$. If it is valid, the $S_{j}$ authenticates the GWN. After receiving the messages $\left\{M_{3},M_{SG},M_{SU},T_{3}\right\}$ from the $S_{j}$, the GWN verifies $M_{SG}^{*}\overset{?}{=}M_{SG}$. If the condition is correct, the GWN authenticates the $S_{j}$. After obtaining the response messages $\left\{M_{4},M_{SU},M_{GU},T_{4}\right\}$ from the GWN, the $U_{i}$ authenticates the GWN. As a result, the $U_{i}$, the $S_{j}$ and the GWN are mutually authenticated because the MA cannot generate exchanged messages $\left\{M_{UG},M_{GS},M_{SG},M_{SU}\right\}$ successfully.

### 7.2. Security Properties

We present the security properties of SLUA-WSN compared to those of the existing schemes [15,37,38,39,40,41]. Table 2 tabulates the security and functionality features of the proposed SLUA-WSN and other existing schemes. According to Table 2, previous schemes [15,37,38,39,40,41] suffer from various attacks, and also their schemes cannot ensure anonymity, untraceability, and mutual authentication. In contrast, SLUA-WSN ensures mutual authentication, anonymity, and untraceability and prevents various attacks. Thus, the proposed SLUA-WSN offers superior security and more functionality features compared with existing schemes.

### 7.3. Formal Security Analysis Using Ban Logic

We perform the BAN logic to demonstrate the mutual authentication of SLUA-WSN. We present notations utilized for BAN logic in Table 3.

#### 7.3.1. Rules of Ban Logic

In the following, the rules of BAN logic are summarized.

**1.**  Message meaning rule:
  $$\frac{N\;|\equiv N\overset{K}{↔}P,\;N⊲{\left\{M\right\}}_{K}}{N\;\left|\equiv P\;\right|∼M}$$
**2.**  Nonce verification rule:
  $$\frac{N\;\left|\equiv \#(M),\;N\;\right|\equiv P\;|∼M}{N\;\left|\equiv P\;\right|\equiv M}$$
**3.**  Jurisdiction rule:
  $$\frac{N\;\left|\equiv P\;\right|⟹M,\;N\;\left|\equiv P\;\right|\equiv M}{N\;|\equiv M}$$
**4.**  Freshness rule:
  $$\frac{N\;|\equiv \#(M)}{N\;|\equiv \#\left(M,W\right)}$$
**5.**  Belief rule:
  $$\frac{N\;|\equiv \left(M,W\right)}{N\;|\equiv M}$$

#### 7.3.2. Goals

We define the following security goals to prove that the proposed SLUA-WSN is capable of performing secure mutual authentication.

**Goal** **1:** $U_{i}|\equiv \left(U_{i}\overset{SK}{⟷}S_{j}\right)$
**Goal** **2:** $S_{j}|\equiv \left(U_{i}\overset{SK}{⟷}S_{j}\right)$
**Goal** **3:** $U_{i}|\equiv S_{j}|\equiv \left(U_{i}\overset{SK}{⟷}S_{j}\right)$
**Goal** **4:** $S_{j}|\equiv U_{i}|\equiv \left(U_{i}\overset{SK}{⟷}S_{j}\right)$

#### 7.3.3. Idealized Forms

The idealized form messages of SLUA-WSN are as below.

**$Msg_{1}$:**  $U_{i}\to GWN$: ${\left(ID_{i},MID_{i},R_{u},T_{1}\right)}_{X_{i}}$
**$Msg_{2}$:**  $GWN\to S_{j}$: ${\left(MID_{i},SID_{j},R_{u},R_{g},T_{2}\right)}_{X_{j}}$
**$Msg_{3}$:**  $S_{j}\to GWN$: ${\left(MID_{i},SID_{j},R_{u},R_{s},T_{3}\right)}_{X_{j}}$
**$Msg_{4}$:**  $GWN\to U_{i}$: ${\left(ID_{MU},R_{g},R_{s},T_{4}\right)}_{X_{i}}$

#### 7.3.4. Assumptions

In the following, the assumptions used in BAN logic are summarized.

**$A_{1}$:**  $GWN|\equiv \#(T_{1})$
**$A_{2}$:**  $GWN|\equiv \#(T_{3})$
**$A_{3}$:**  $S_{j}|\equiv \#\left(T_{2}\right)$
**$A_{4}$:**  $U_{i}|\equiv \#\left(T_{4}\right)$
**$A_{5}$:**  $GWN|\equiv (GWN\overset{X_{j}}{⟷}S_{j})$
**$A_{6}$:**  $S_{j}|\equiv \left(GWN\overset{X_{j}}{⟷}S_{j}\right)$
**$A_{7}$:**  $U_{i}|\equiv \left(U_{i}\overset{X_{i}}{⟷}GWN\right)$
**$A_{8}$:**  $GWN|\equiv (U_{i}\overset{X_{i}}{⟷}GWN)$
**$A_{9}$:**  $U_{i}|\equiv S_{j}\Rightarrow \left(U_{i}\overset{SK}{⟷}S_{j}\right)$
**$A_{10}$:**  $S_{j}|\equiv U_{i}\Rightarrow \left(U_{i}\overset{SK}{⟷}S_{j}\right)$

#### 7.3.5. Proof Using Ban Logic

The BAN logic proof then proceeds as below.

**Step** **1:** According to $Msg_{1}$, we could get the following,
  $$\left(S_{1}\right):GWN⊲{\left(ID_{i},MID_{i},R_{u},T_{1}\right)}_{X_{i}}$$
**Step** **2:** Using $S_{1}$ and $A_{8}$ with “message meaning rule”, the following is obtained,
  $$\left(S_{2}\right):GWN|\equiv MU|∼{\left(ID_{i},MID_{i},R_{u},T_{1}\right)}_{X_{i}}$$
**Step** **3:** Using $S_{2}$ and $A_{1}$ with “freshness rule”, the following is obtained,
  $$\left(S_{3}\right):GWN|\equiv \#{\left(ID_{i},MID_{i},R_{u},T_{1}\right)}_{X_{i}}$$
**Step** **4:** From $S_{2}$ and $S_{3}$ with “nonce verification rule”, we could get
  $$\left(S_{4}\right):GWN|\equiv U_{i}|\equiv {\left(ID_{i},MID_{i},R_{u},T_{1}\right)}_{X_{i}}$$
**Step** **5:** According to $Msg_{2}$, we could get
  $$\left(S_{5}\right):S_{j}⊲{\left(MID_{i},SID_{j},R_{u},R_{g},T_{2}\right)}_{X_{j}}$$
**Step** **6:** Using the $S_{5}$ and $A_{6}$ with “message meaning rule”, the following is obtained,
  $$\left(S_{6}\right):S_{j}|\equiv GWN|∼{\left(MID_{i},SID_{j},R_{u},R_{g},T_{2}\right)}_{X_{j}}$$
**Step** **7:** Now, using $S_{6}$ and $A_{3}$ with “freshness rule”, we could get
  $$\left(S_{7}\right):S_{j}|\equiv \#{\left(MID_{i},SID_{j},R_{u},R_{g},T_{2}\right)}_{X_{j}}$$
**Step** **8:** Utilizing $S_{6}$ and $S_{7}$ with “nonce verification rule”, the following is obtained,
  $$\left(S_{8}\right):S_{j}|\equiv GWN|\equiv {\left(MID_{i},SID_{j},R_{u},R_{g},T_{2}\right)}_{X_{j}}$$
**Step** **9:** According to $Msg_{3}$, we could get the following,
  $$\left(S_{9}\right):GWN⊲{\left(MID_{i},SID_{j},R_{u},R_{s},T_{3}\right)}_{X_{j}}$$
**Step** **10:** Using $S_{9}$ and $A_{5}$ with “message meaning rule”, the following is obtained,
  $$\left(S_{10}\right):GWN|\equiv S_{j}|∼{\left(MID_{i},SID_{j},R_{u},R_{s},T_{3}\right)}_{X_{j}}$$
**Step** **11:** Using $S_{10}$ and $A_{2}$ with “freshness rule”, the following is obtained,
  $$\left(S_{11}\right):GWN|\equiv \#{\left(MID_{i},SID_{j},R_{u},R_{s},T_{3}\right)}_{X_{j}}$$
**Step** **12:** From $S_{10}$ and $S_{11}$ with “nonce verification rule”, we could get
  $$\left(S_{12}\right):GWN|\equiv U_{i}|\equiv {\left(MID_{i},SID_{j},R_{u},R_{s},T_{3}\right)}_{X_{j}}$$
**Step** **13:** According to $Msg_{4}$, we could get the following,
  $$\left(S_{13}\right):U_{i}⊲{\left(ID_{MU},R_{g},R_{s},T_{4}\right)}_{X_{i}}$$
**Step** **14:** Using $S_{13}$ and $A_{7}$ with “message meaning rule”, the following is obtained,
  $$\left(S_{14}\right):U_{i}|\equiv GWN|∼{\left(ID_{MU},R_{g},R_{s},T_{4}\right)}_{X_{i}}$$
**Step** **15:** Using $S_{14}$ and $A_{4}$ with “freshness rule”, the following is obtained,
  $$\left(S_{15}\right):U_{i}|\equiv \#{\left(ID_{MU},R_{g},R_{s},T_{4}\right)}_{X_{i}}$$
**Step** **16:** From $S_{14}$ and $S_{15}$ with “nonce verification rule”, we could get
  $$\left(S_{16}\right):U_{i}|\equiv GWN|\equiv {\left(ID_{MU},R_{g},R_{s},T_{4}\right)}_{X_{i}}$$
**Step** **17:** Because $SK=h(R_{u}||R_{s})$, according to $S_{12}$ and $S_{16}$, the following is obtained,
  $$\left(S_{17}\right):U_{i}|\equiv S_{j}|\equiv \left(U_{i}\overset{SK}{⟷}S_{j}\right)\;\;\left(\mathrm{Goal}3\right)$$
**Step** **18:** Because $SK=h(R_{u}||R_{s})$, according to $S_{4}$ and $S_{8}$, we could get
  $$\left(S_{18}\right):S_{j}|\equiv U_{i}|\equiv \left(U_{i}\overset{SK}{⟷}S_{j}\right)\;\;\left(\mathrm{Goal}4\right)$$
**Step** **19:** From $A_{9}$ and $S_{17}$, the following is obtained,
  $$\left(S_{19}\right):U_{i}|\equiv \left(U_{i}\overset{SK}{⟷}S_{j}\right)\;\;\left(\mathrm{Goal}1\right)$$
**Step** **20:** Using $A_{10}$ and $S_{18}$, the following is obtained,
  $$\left(S_{20}\right):S_{j}|\equiv \left(U_{i}\overset{SK}{⟷}S_{j}\right)\;\;\left(\mathrm{Goal}2\right)$$

According to Goals 1–4, we prove that the proposed SLUA-WSN ensures secure mutual authentication among $U_{i}$, GWN, and $S_{j}$.

### 7.4. Formal Security Analysis Using Ror Model

We perform the ROR model [17] to evaluate the session key (SK) security of SLUA-WSN from the malicious attacker MA. Initially, we introduce the ROR model [17] before performing the analysis of SK security for SLUA-WSN.

In the ROR model, the malicious attacker MA interacts with the $P_{MA}^{t}$, the $t^{th}$ instance of the executing participant. Furthermore, there are three participants—the user $P_{U_{i}}^{t_{1}}$, gateway $P_{GWN}^{t_{2}}$, and sensor $P_{S_{j}}^{t_{3}}$—where $P_{U_{i}}^{t_{1}}$, $P_{GWN}^{t_{2}}$, and $P_{S_{j}}^{t_{3}}$ are instances $t_{1}^{th}$ of $U_{i}$, $t_{2}^{th}$ of GWN, and $t_{3}^{th}$ of $S_{j}$, respectively. In Table 4, we define various queries for ROR model to evaluate security analysis such as Execute, CorruptSC, Reveal, Send, and Test. Furthermore, an one-way hash function h(·) is modeled as a random oracle Hash. We utilize Zipf’s law [48] to evaluate SK security of SLUA-WSN.

**Theorem** **1.** *If $Adv_{MA}$ denotes the advantage function of the MA in violating SK security of SLUA-WSN. After that, we can derive the following.*

$$Adv_{MA}\leq \frac{q_{h}^{2}}{\left|Hash\right|}+2\left\{C\cdot q_{send}^{s},\frac{q_{s}}{2^{l_{b}}}\right\}$$

*where $q_{h}$, |Hash|, and $q_{send}$ are the number of Hash, the range space of Hash, and the number of Send queries, respectively. Furthermore, C, $l_{b}$, and s are parameters used in Zipf’s laws [48].*

**Proof** **1.** We define the following four games, namely, $G_{i}$ (i∈[0,3]). We indicate that $Succ_{i}$ is the probability of MA winning the $G_{i}$. All $G_{i}$ are described in detail as shown below.

- **Game**$G_{0}$: The first game $G_{0}$ is considered as an passive attack executed from the MA in the proposed protocol *P*, as the bit *C* is guessed randomly at the beginning of $G_{0}$. According to this game, the following is obtained.

(1) $$Adv_{MA}=\left|2\cdot Pr\left[Succ_{0}\right]-1\right|$$

- **Game**$G_{1}$: This $G_{1}$ considers the scenario where MA simulates the eavesdropping attack in which the transmitted messages are intercepted during the authentication process using the Execute query. After eavesdropping transmitted messages, the MA performs the Reveal and Test queries to verify whether it is the SK or a random number. The MA needs the secret parameters, such as $R_{u}$, $R_{s}$, $X_{i}$, and $X_{j}$, to derive $SK=h(R_{u}||R_{s})$. Thus, the MA does not at all help in increasing the $G_{1}$’s winning probability by eavesdropping on the transmitted messages. According to this game, the following is obtained.

(2) $$Pr\left[Succ_{1}\right]=Pr\left[Succ_{0}\right]$$

- **Game**$G_{2}$: $G_{2}$ is modeled as an active attack, where the simulations of the Send and Hash oracles are included. In $G_{2}$, the MA can eavesdrop all exchanged messages $\left\{M_{1},MID_{i},CID_{i},M_{UG},T_{1}\right\}$, $\left\{M_{2},MID_{i},M_{GS},T_{2}\right\}$, $\left\{M_{3},M_{SG},M_{SU},T_{3}\right\}$, and $\left\{M_{4},M_{SU},M_{GU},T_{4}\right\}$ during the authentication and key agreement process. However, all exchanged messages are safeguarded using the hash function h(·). Furthermore, the random numbers $R_{u}$ and $R_{s}$ are not derived from the intercepted exchanged messages because the random numbers are protected by hash function h(·). By applying the birthday paradox [49], we can derive the following.

(3) $$|Pr\left[Succ_{2}\right]-Pr\left[Succ_{1}\right]|\leq \frac{q_{h}^{2}}{2|Hash|}$$

- **Game**$G_{3}$: $G_{3}$ is simulated using CorruptSC query. In this game, the MA is able to extract the secret credentials $\left\{Q_{i},W_{i},MID_{i}\right\}$ from a smartcard’s memory using the power analysis attack. Generally, a user utilizes the low-entropy password. Using SC’s stored secret credentials $\left\{Q_{i},W_{i},MID_{i}\right\}$, the MA may try to extract the password $PW_{i}$ by performing a password guessing attack. However, in the proposed protocol, the MA cannot obtain password $PW_{i}$ of the legitimate user correctly through the Send query without GWN’s master key $K_{GWN}$ and secret parameter $X_{i}$. Furthermore, the probability of guessing the biometric secret key $b_{i}$ of $l_{b}$ bits by the MA is approximately $\frac{1}{2^{l_{b}}}$. Thus, the $G_{2}$ and $G_{3}$ are indistinguishable if biometric/password guessing attacks are not present. Consequently, by applying Zipf’s law [48], the following is obtained.

(4) $$|Pr\left[Succ_{3}\right]-Pr\left[Succ_{2}\right]|\leq max\left\{C\cdot q_{send}^{s},\frac{q_{s}}{2^{l_{b}}}\right\}$$

When all the games are executed, the MA should guess the correct bit *c*. Consequently, we can obtain the following result.

(5) $$Pr\left[Succ_{3}\right]=\frac{1}{2}$$

By applying Equations (1), (2), and (5), the following result is obtained.

(6) $$\begin{array}{cc} \frac{1}{2}Adv_{MA} & =|Pr\left[Succ_{0}\right]-\frac{1}{2}|\\ \; & =|Pr\left[Succ_{1}\right]-\frac{1}{2}|\\ \; & =|Pr\left[Succ_{1}\right]-Pr\left[Succ_{3}\right]| \end{array}$$

By applying Equations (4)–(6), the following result is obtained, utilizing the triangular inequality.

(7) $$\begin{array}{cc} \frac{1}{2}Adv_{U_{A}} & =|Pr\left[Succ_{1}\right]-Pr\left[Succ_{3}\right]|\\ \; & \leq |Pr\left[Succ_{1}\right]-Pr\left[Succ_{2}\right]|\\ \; & \;+|Pr\left[Succ_{2}\right]-Pr\left[Succ_{3}\right]|\\ \; & \leq \frac{q_{h}^{2}}{2|Hash|}+max\left\{C\cdot q_{send}^{s},\frac{q_{s}}{2^{l_{b}}}\right\} \end{array}$$

As a result, multiplying both sides of Equation (7) by a factor of two, the following result is obtained.

$$Adv_{MA}\leq \frac{q_{h}^{2}}{\left|Hash\right|}+2max\left\{C\cdot q_{send}^{s},\frac{q_{s}}{2^{l_{b}}}\right\}$$

□

### 7.5. AVISPA Simulation

We perform the AVISPA simulation tool [18,19] to prove the security of SLUA-WSN against MITM and replay attacks. To perform the AVISPA simulation, the environment and session of the protocol must be implemented utilizing the High-Level Protocols Specification Language (HLPSL) [50].

#### 7.5.1. HLPSL Specification

**Figure 7:**
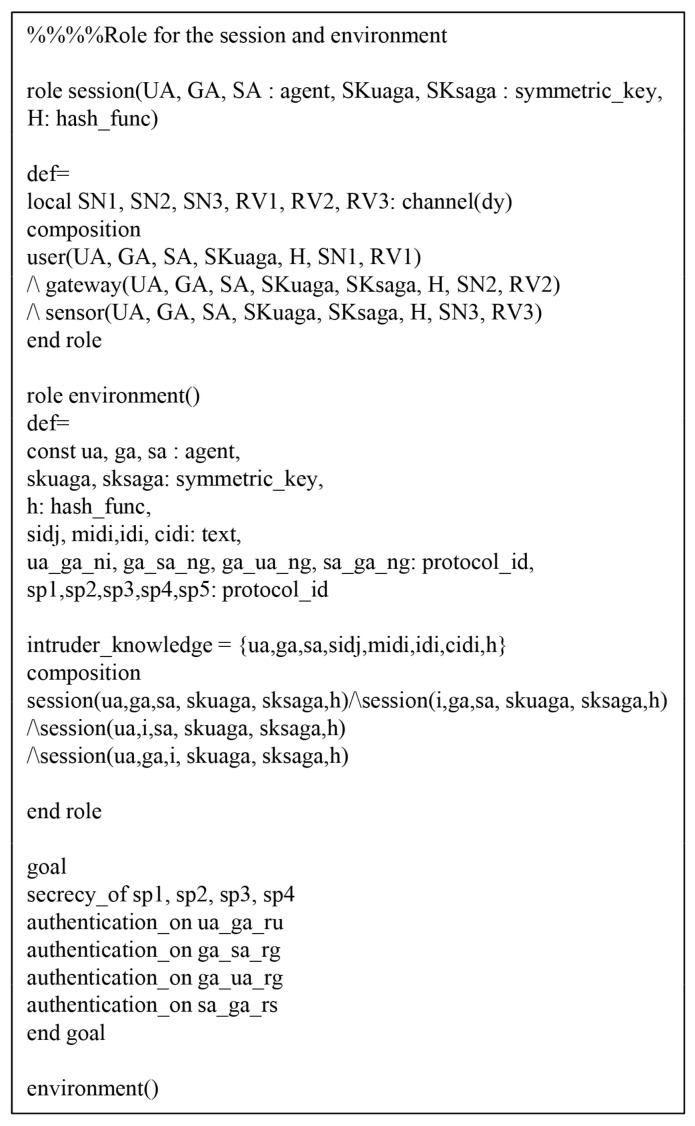
High-Level Protocols Specification Language (HLPSL) syntax for session and environment.

Referring to HLPSL, we consider three roles: the $U_{i}$, the GWN, and the $S_{j}$. We present the environment and session using HLPSL in Figure 7, which consists of the security goals.

**Figure 8:**
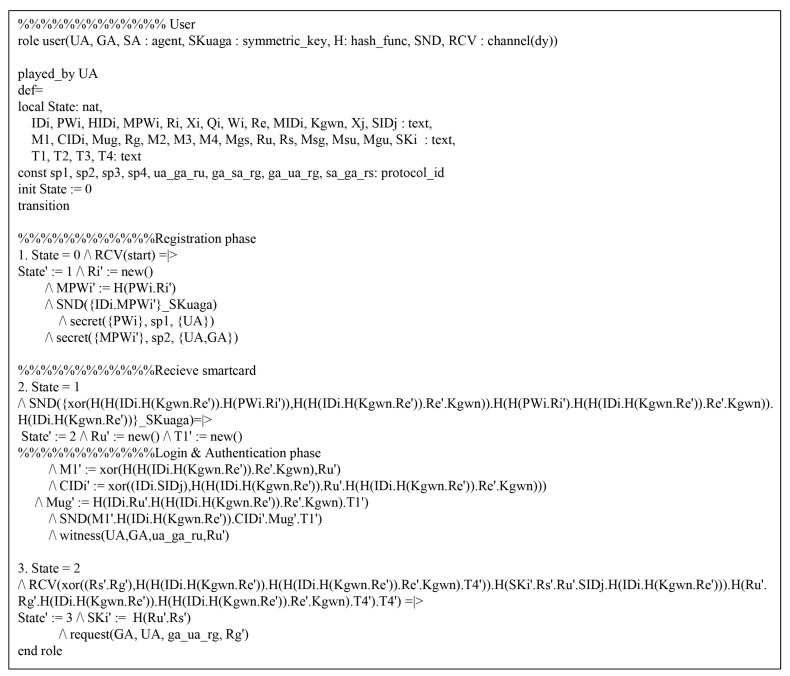
HLPSL syntax for $U_{i}$.

**Figure 9:**
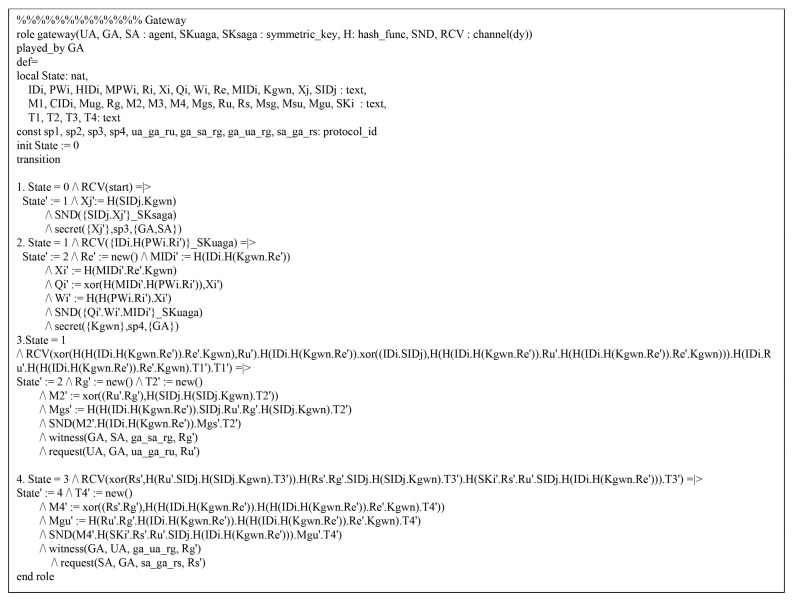
HLPSL syntax for GWN.

**Figure 10:**
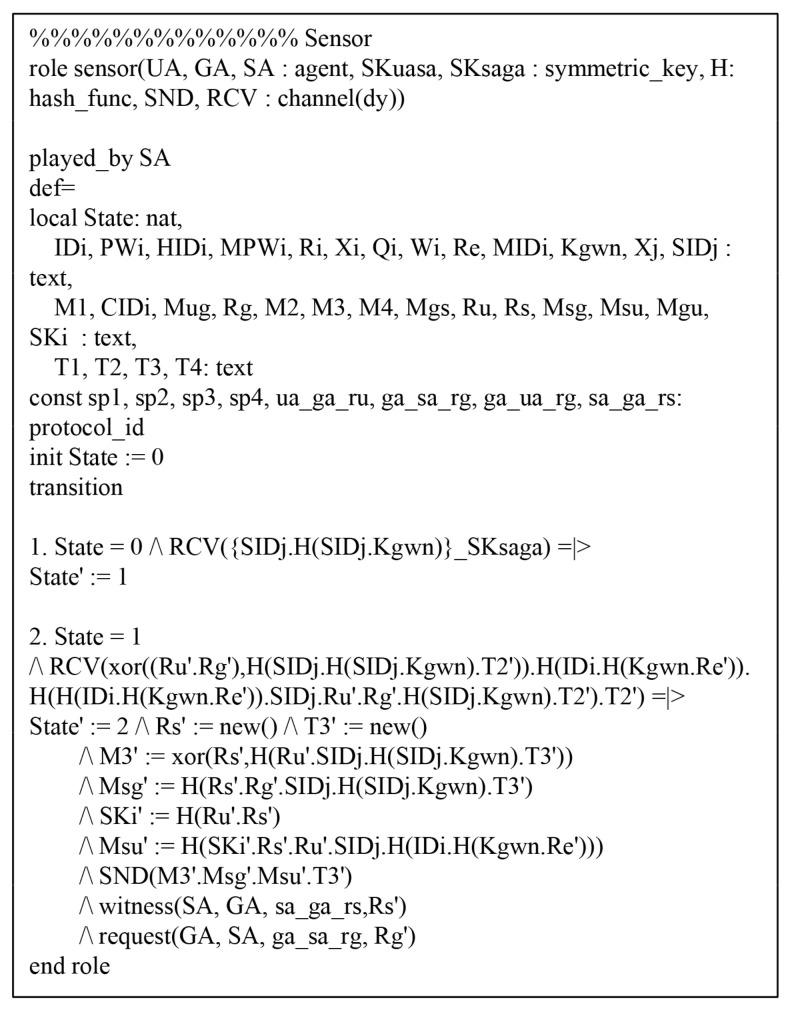
HLPSL syntax for $S_{j}$.

In Figure 8, the $U_{i}$ initially receives the message and updates the state value from 1 to 2. After that, $U_{i}$ transmits the registration request message $\left\{ID_{i},MPW_{i}\right\}$ to GWN over a secure channel. Then, $U_{i}$ receives the {smartcard} from GWN and $U_{i}$ changes the state value from 1 to 2. In the authentication process, the $U_{i}$ should send an authentication request message $\left\{M_{1},MID_{i},CID_{i},M_{UG},T_{1}\right\}$ to GWN over a public channel. Thus, the $U_{i}$ declares $witness(UA,GA,ua\_ga\_ru,RU^{{}^{\prime }})$ from the GWN, and then changes the state value from 2 to 3. Then, $U_{i}$ receives the authentication response messages $\left\{M_{4},M_{SU},M_{GU},T_{4}\right\}$ from the GWN. Finally, $U_{i}$ checks $M_{GU}^{*}\overset{?}{=}M_{GU}$ and $M_{SU}^{*}\overset{?}{=}M_{SU}$. If it is correct, the $U_{i}$, GWN, and $S_{j}$ are mutually authenticated successfully. In addition, the HLPSL specification roles of GWN and $S_{j}$ are similarly defined. Figure 9 and Figure 10 show the role specification of the GWN and $S_{j}$.

#### 7.5.2. AVISPA Simulation Result

**Figure 11:**
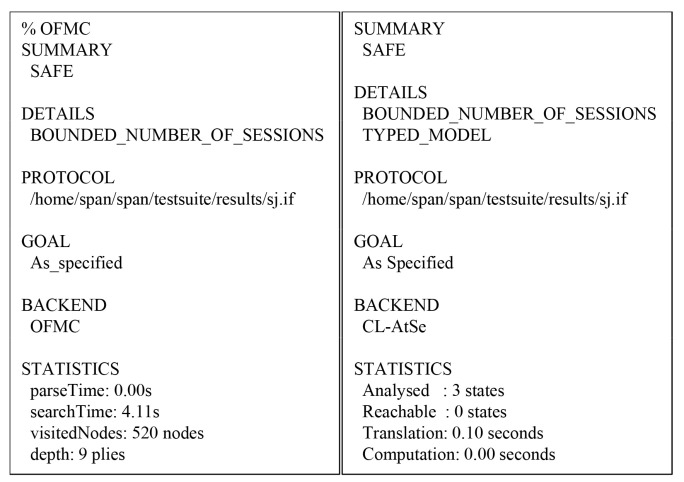
AVISPA simulation results using On-the-Fly Model Checker (OFMC) and Constraint-Logic-based ATtack SEarcher (CL-AtSe).

We present the AVISPA simulation result to demonstrate the security of the SLUA-WSN utilizing On-the-Fly Model Checker (OFMC) and Constraint-Logic-based ATtack SEarcher (CL-AtSe) back-ends. The OFMC and CL-AtSe back-ends verify whether a legitimate entity is able to execute the protocol by searching for a passive attacker. In addition, CL-AtSe and OFMC back-ends check that the SLUA-WSN is secure against the replay and MITM attacks based on the DY model. According to Figure 11, the proposed SLUA-WSN is secure against MITM and replay attacks. Moreover, the result of OFMC validation shows that the search time was 4.11 s for visiting 520 nodes, and the result of the CL-AtSe validation analyzed three states and the translation time was 0.10 s. We provide similar AVISPA simulation results as adopted in [51,52,53,54,55].

## 8. Performance Analysis

We evaluate the performance of SLUA-WSN in terms of the computation, communication, and storage overheads. We also compare SLUA-WSN with other existing schemes [15,37,38,39,40,41].

### 8.1. Computation Overheads

This section compares the computation overhead associated with the SLUA-WSN to those of related schemes [15,37,38,39,40,41] during the authentication process. We analyzed utilizing the following parameters to evaluate the computation overhead. Referring to the work in [15], $T_{m}$, $T_{R}$, $T_{S}$, and $T_{h}$ denote the execution time for point multiplication (≈7.3529 ms), rep operation (≈7.3529 ms), symmetric encryption/decryption (≈0.1303 ms), and hash function (≈0.0004 ms), respectively. The execution time of XOR operation is not included because it is negligible. In Table 5, we show the results of the computation overhead comparison. Consequently, SLUA-WSN provides a more efficient computation cost compared with the other existing schemes [15,37,38,39,40,41].

### 8.2. Communication Overheads

We compare the communication cost with the related schemes [15,37,38,39,40,41]. Referring to the work in [15], we assume that the hash function, a timestamp, an identity, a random nonce, and a prime *p* are 160 bits, 32 bits, 32 bits, 128 bits, and 160 bits, respectively. In addition, we consider that an ECC of 160 bits has a security level equivalent to that of the 1024-bit RSA [56]. The block size of plaintext/ciphertext for the AES algorithm is 128 bits [57]. In the authentication process of SLUA-WSN, the exchanged messages $\left\{M_{1},MID_{i},CID_{i},M_{UG},T_{1}\right\}$, $\left\{M_{2},MID_{i},M_{GS},T_{2}\right\}$, $\left\{M_{3},M_{SG},M_{SU},T_{3}\right\}$, and $\left\{M_{4},M_{SU},M_{GU},T_{4}\right\}$ require (160 + 160 + 160 + 160 + 32 = 672 bits), (160 + 160 + 160 + 32 = 512 bits), (160 + 160 + 160 + 32 = 512 bits), and (160 + 160 + 160 + 32 = 512 bits), respectively. In Table 6, we present the results of the communication overhead comparison. Thus, SLUA-WSN has a more efficient communication cost compared with other related schemes [15,37,38,39,40,41].

### 8.3. Storage Overheads

We compare the storage costs with the related schemes [15,37,38,39,40,41]. We first define that the hash, identity, timestamp, random nonce, ECC algorithm, RSA algorithm, and AES algorithm are 20, 4, 4, 16, 20, 128, and 16 bytes, respectively, and the prime *p* in $E_{p}\left(a,b\right)$ is 20 bytes. In the proposed SLUA-WSN, stored messages $\left\{Q_{i},W_{i},MID_{i}\right\}$ and $\left\{r_{g}\right\}$ require (20 + 20 + 20 = 60 bytes) and (20 bytes), respectively. Although the storage costs of the proposed SLUA-WSN are somewhat higher than Mo and Chen’s scheme [15], it provides better security and efficiency than the other related schemes [15,37,38,39,40,41]. Table 7 shows the analysis results of storage overhead compared to related schemes.

## 9. Conclusions

In this paper, we proved that Mo and Chen’s scheme suffers from various security flaws, such as session key exposure and masquerade attacks, and does not provide anonymity, untraceability, and authentication. We proposed a secure and lightweight user authentication protocol in WSN environments utilizing biometric and secret parameters to resolve the security drawbacks of Mo and Chen’s protocol. SLUA-WSN prevents various attacks, including sensor node capture, masquerade, and privileged insider attacks. We demonstrated that the proposed SLUA-WSN ensures secure mutual authentication between $U_{i}$, GWN, and $S_{j}$ by performing BAN logic. We also proved the security of SLUA-WSN by performing the formal security analysis such as the ROR model and AVISPA simulation. We compared the performance of SLUA-WSN in terms of computation, communication, and storage overheads with existing schemes. Consequently, the proposed SLUA-WSN provided a great improvement in terms of the security level compared with three-factor-based related schemes and also preserved the low computation and communication overheads using only hash and XOR operations. Therefore, the proposed SLUA-WSN provides superior security and efficiency than related schemes and is suitable for practical WSN environments.

## Figures and Tables

**Table 1:** Notations.

Notation	Description
$U_{i}$	User
GWN	Gateway node
$S_{j}$	Sensor node
$ID_{i}$	$U_{i}$’s identity
$PW_{i}$	$U_{i}$’s password
$SID_{j}$	$S_{j}$’s identity
$K_{GWN}$	Master key of GWN
$X_{pub}$	Public key of GWN
$X_{j}$	Secret key of $S_{j}$
$E/F_{p}$	Elliptic curve *E* defined on the finite field $F_{p}$ with order *p*
*G*	A group for an elliptic curve
*P*	The generator of *G*
$E_{k}/D_{k}$	Symmetric key encryption/decryption
SK	Session key
$T_{i}$	Timestamp
BIO	Biometric of $U_{i}$
h(·)	Hash function
⊕	XOR operation
||	Concatenation operation

**Table 2:** Security property comparison.

Security Properties	Wu et al. [37]	Wang et al. [38]	Li et al. [39]	Li et al. [40]	Lu et al. [41]	Mo and Chen [15]	Ours
Three-factor security	×	∘	×	∘	×	∘	∘
Masquerade attack	×	∘	×	×	×	×	∘
Replay attack	×	∘	×	×	∘	∘	∘
Privileged insider attack	∘	×	∘	×	∘	∘	∘
Sensor node capture attack	∘	∘	∘	∘	∘	∘	∘
Man-in-the-middle attack	∘	∘	×	×	∘	∘	∘
User anonymity	∘	∘	∘	∘	∘	×	∘
Untraceability	∘	∘	∘	∘	∘	×	∘
Mutual authentication	∘	∘	∘	∘	∘	×	∘

∘: it supports security properties; ×: it does not support security properties.

**Table 3:** Notations used for BAN logic.

Notation	Description
N|≡M	*N* **believes** *M*
#M	*M* is updated and **fresh**
N⊲M	*N* **sees** *M*
N|∼M	*N* once **said** *M*
N⇒M	*N***controls** that *M*
$<M{>}_{W}$	*M* is **combined** with *W*
${\left\{M\right\}}_{K}$	*M* is **encrypted** utilizing symmetric key *K*
$N\overset{K}{↔}P$	*N* and *P* share a **shared secret key** *K*
SK	**Session key** used in communication session

**Table 4:** Queries of the Real-or-Random (ROR) model.

Query	Description
$Execute(P_{U_{i}}^{t_{1}},P_{GWN}^{t_{2}},P_{S_{j}}^{t_{3}})$	Execute denotes that MA performs the passive attack by eavesdropping transmitted messages between legitimate participants over an insecure channel.
$CorruptSC(P_{U_{i}}^{t_{1}})$	CorruptSC is modeled that the smartcard stolen attack, in which the MA can extract the secret credentials stored in the smartcard.
$Send(P^{t},M)$	Using this query, the MA can transmit a message *M* to the instance $P^{t}$ and also can receive accordingly.
$Test(P^{t})$	Test corresponds to the semantic security of the SK between $U_{i}$ and $S_{j}$ following the indistinguishability style in the ROR model [17]. In this query, an unbiased coin *c* is flipped prior to the starting of the experiment. If the MA performs Test query and the corresponding SK is fresh, and then $P^{t}$ returns SK when c=1 after running Test query, SK is new or a random number when c=0; otherwise, it delivers a null value (⊥).
$Reveal(P^{t})$	Using this query, the MA reveals the current SK generated by its partner to an adversary MA.

**Table 5:** Computation overheads comparison.

Schemes	User	Gateway	Sensor node	Total	Computation overhead
Wu et al. [37]	$11T_{h}+T_{R}+2T_{m}$	$10T_{h}$	$3T_{h}+2T_{m}$	$24T_{h}+T_{R}+4T_{m}$	36.77 ms
Wang et al. [38]	$10T_{h}+T_{R}+3T_{m}$	$13T_{h}+T_{m}$	$6T_{h}+2T_{m}$	$29T_{h}+T_{R}+6T_{m}$	51.48 ms
Li et al. [39]	$8T_{h}+T_{R}+2T_{m}$	$9T_{h}+T_{m}$	$4T_{h}$	$21T_{h}+T_{R}+3T_{m}$	29.42 ms
Li et al. [40]	$12T_{h}+3T_{m}$	$8T_{h}+T_{m}$	$4T_{h}+2T_{m}$	$24T_{h}+6T_{m}$	44.13 ms
Lu et al. [41]	$7T_{h}+T_{R}+3T_{m}+T_{S}$	$6T_{h}+T_{m}+T_{S}$	$2T_{h}+2T_{m}+2T_{S}$	$15T_{h}+T_{R}+6T_{m}+4T_{S}$	51.99 ms
Mo and Chen [15]	$12T_{h}+T_{R}+2T_{m}$	$10T_{h}+T_{S}$	$5T_{h}+2T_{m}+T_{S}$	$27T_{h}+T_{R}+4T_{m}+2T_{S}$	37.03 ms
Ours	$11T_{h}+T_{R}$	$11T_{h}$	$6T_{h}$	$28T_{h}+T_{R}$	7.36 ms

**Table 6:** Communication overheads comparison.

Schemes	Communication Overhead	Number of Messages
Wu et al. [37]	3072 bits	4 messages
Wang et al. [38]	2368 bits	4 messages
Li et al. [39]	2496 bits	4 messages
Li et al. [40]	2880 bits	4 messages
Lu et al. [41]	2880 bits	3 messages
Mo and Chen [15]	3328 bits	4 messages
Ours	2208 bits	4 messages

**Table 7:** Storage overheads comparison.

Schemes	Stored Message (Smart Card/mobile Device)	Stored Message (Gateway Node)
Wu et al. [37]	$B_{1},B_{2},P_{bi}\approx 56$ bytes	$ID_{i}\approx 4$ bytes
Wang et al. [38]	$A_{i},B_{i},n_{0},Y,P\approx 100$ bytes	$ID_{i},r_{i}\approx 20$ bytes
Li et al. [39]	$\alpha ,\delta ,A_{i},B_{i},X\approx 92$ bytes	$ID_{i}\approx 4$ bytes
Li et al. [40]	$A_{i},B_{i},E_{i},X,f,n_{0},r\approx 108$ bytes	$ID_{i},k_{i}\approx 20$ bytes
Lu et al. [41]	$RPW_{i},f_{i},v_{i}\approx 56$ bytes	$K_{j}\approx 20$ bytes
Mo and Chen [15]	$RID_{i},f_{i},\tau \approx 56$ bytes	$K_{j}\approx 20$ bytes
Ours	$Q_{i},W_{i},MID_{i}\approx 60$ bytes	$r_{g}\approx 20$ bytes
